# Crystal structure of an extracellular superoxide dismutase from *Onchocerca volvulus* and implications for parasite-specific drug development

**DOI:** 10.1107/S2053230X22005350

**Published:** 2022-05-27

**Authors:** Amr Moustafa, Markus Perbandt, Eva Liebau, Christian Betzel, Sven Falke

**Affiliations:** a Laboratory for Structural Biology of Infection and Inflammation, University of Hamburg, c/o DESY, Building 22a, Notkestrasse 85, 22607 Hamburg, Germany; b Biochemistry Department, Faculty of Veterinary Medicine, Zagazig University, Zagazig 44519, Egypt; c Institut für Zoophysiologie, Westfälische Wilhelms-Universität Münster, Schlossplatz 8, 48143 Münster, Germany

**Keywords:** X-ray crystallography, Cu/Zn superoxide dismutases, metal ion coordination, *Onchocerca volvulus*, parasites, docking, drug targets

## Abstract

Superoxide dismutases (SODs) are metalloproteins that are responsible for the dismutation of superoxide anion radicals. SODs are consequently protective against oxidative damage to cellular components. Among other protective mechanisms, the filarial parasite *Onchocerca volvulus* has a well developed defense system to scavenge toxic free radicals using SODs during migration and sojourning of the microfilariae and adult worms in the human body. *O. volvulus* is responsible for the neglected disease onchocerciasis or ‘river blindness’. In the present study, an extracellular Cu/Zn-SOD from *O. volvulus* (*Ov*EC-SOD) was cloned, purified and crystallized to obtain structural insight into an attractive drug target with the potential to combat onchocerciasis. The recombinant *Ov*EC-SOD forms a dimer and the protein structure was solved and refined to 1.55 Å resolution by X-ray crystallography. Interestingly, a sulfate ion supports the coordination of the conserved copper ion. The overall protein shape was verified by small-angle X-ray scattering. The enzyme shows a different surface charge distribution and different termini when compared with the homologous human SOD. A distinct hydrophobic cleft to which both protomers of the dimer contribute was utilized for a docking approach with compounds that have previously been identified as SOD inhibitors to highlight the potential for individual structure-based drug development.

## Abstract 2

An extracellular Cu/Zn superoxide dismutase from *Onchocerca volvulus*, the causative agent of human onchocerciasis, was purified and crystallized and the structure was solved at 1.55 Å resolution. The solution structure of the dimeric protein was verified using small-angle X-ray scattering. Initial docking studies utilizing previously identified superoxide dismutase inhibitors indicate the potential for future drug development targeting structural features outside the active site.

## 1. Introduction

The filarial parasite *Onchocerca volvulus* is the causative agent of human onchocerciasis, an infectious disease characterized by skin lesions, acute and chronic dermatitis, and depigmentation (Brattig *et al.*, 1994 ▸). It is the second leading cause of infectious blindness worldwide. At least 220 million people are estimated to have required preventive chemotherapy against onchocerciasis. 99% of infected people live in Africa and 1.15 million people are visually impaired or blind according to the World Health Organization (World Health Organization, 2022 ▸). Onchocerciasis causes chronic disability with long-term complications and socio-economic problems, particularly in developing countries. A number of strategies to control onchocerciasis have aimed at targeting the transmitting vector, *i.e.* the blackfly, and/or fighting *O. volvulus* in the human host itself. Among various approaches, the drug ivermectin has frequently been used (Basáñez *et al.*, 2008 ▸). However, ivermectin is only effective against microfilariae (Borsboom *et al.*, 2003 ▸), has adverse effects and emerging resistance has also been reported due to its massive utilization (Keiser *et al.*, 2002 ▸; Osei-Atweneboana *et al.*, 2007 ▸). Hence, there is an urgent need for the development of drugs that also target adult worms since they resume the production of microfilariae (Churcher *et al.*, 2009 ▸). Alternative treatments using medicinal plants have been also put in place, using *Onchocerca ochengi* and *Caenorhabditis elegans* as model organisms (Cho-Ngwa *et al.*, 2010 ▸; Ndjonka *et al.*, 2011 ▸). Moreover, substantial work has focused on targeting *Wolbachia* bacteria, which coexist in symbiosis with filarial worms (Harcus *et al.*, 2004 ▸; Wanji *et al.*, 2009 ▸). Although these attempts have been successful in decreasing the number of cases, onchocerciasis has by no means been eliminated and continues to be a major public health concern. One additional approach focuses on the identification of excretory/secretory products (ESPs) that are essentially involved in parasite–host interaction. Their diversity and extracellular accessibility render them attractive drug targets (Hewitson *et al.*, 2008 ▸; Lustigman *et al.*, 2002 ▸). The secretion of antioxidant enzymes by parasites is thought to predominantly protect them against toxic reactive oxygen species (ROS) released by immune effector cells as a host defense mechanism (Hewitson *et al.*, 2008 ▸). *O. volvulus* responds to ROS by producing antioxidant enzymes such as thioredoxin peroxidase (Chandrashekar *et al.*, 1998 ▸), glutathione *S*-transferases (Liebau *et al.*, 2008 ▸) and superoxide dismutases (SODs; Henkle-Dührsen *et al.*, 1997 ▸; Lizotte-Waniewski *et al.*, 2000 ▸). SODs are metalloenzymes that catalyse the disproportion redox reaction of superoxide anions to oxygen and hydrogen peroxide, *i.e.* 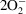 + 2H^+^ → O_2_ + H_2_O_2_. In addition to oxidative stress, this is also relevant for peroxide signaling (Montllor-Albalate *et al.*, 2019 ▸). Since H_2_O_2_ is a rather inert and small oxidant, it can freely diffuse through cell membranes and possesses multiple physiological effects, as discussed elsewhere (Storz & Imlayt, 1999 ▸; Rahbari *et al.*, 2017 ▸). SODs are distinguished based on their cellular localization and the metal cofactor(s) in their active sites. All SOD subgroups have been characterized in humans in great detail (Petersen *et al.*, 2004 ▸, 2008 ▸), as well as, for example, in fungi (Robinett *et al.*, 2018 ▸; Mohsin *et al.*, 2021 ▸) and to a limited extent in nematodes (Dabir *et al.*, 2008 ▸; Henkle-Dührsen *et al.*, 1994 ▸; James *et al.*, 1994 ▸; Ou *et al.*, 1995 ▸). A number of copper(II) complexes are capable of mimicking SOD activity (Siqueira *et al.*, 2020 ▸). Nanocomposite-based materials with SOD activity are being considered for pharmaceutical use in the treatment of stress-related diseases (Pavlovic *et al.*, 2021 ▸). Some high-resolution structures of SODs have been determined, among which is the recently reported structure of a Cu/Zn-SOD from the fungus *Chaetomium thermophilum* (Mohsin *et al.*, 2021 ▸).

The first characterization of a SOD from *O. volvulus* was reported by Henkle *et al.* (1991 ▸), but additional SOD activity was found in *in vitro* culture supernatants of *Onchocerca* microfilariae and adult worms (James *et al.*, 1994 ▸). The suggestion that there is another secretory or excretory form of this enzyme was supported by a study that detected a SOD in larval and adult stages (Henkle-Dührsen *et al.*, 1997 ▸). It was first predicted that the individual N-terminal signal peptide (SP) of this extracellular SOD (*Ov*EC-SOD; EC 1.15.1.1) is cleaved off between Asn42 and Gly43 of the preprocessed protein (James *et al.*, 1994 ▸). Although computational tools for the detection of SPs have continuously improved, the detection of cleavage sites remains challenging. This is especially critical when preparing the recombinant production of putative ESPs for structural studies.

In terms of our investigations, we successfully designed a soluble *Ov*EC-SOD construct starting from Gly43 of the preprocessed protein. The structure of *Ov*EC-SOD was solved at 1.55 Å resolution, comprising a homodimer with 156 residues and one copper and one zinc ion in the active site of each protomer. Despite the conserved fold, the overall sequence identities compared with human cytosolic and extracellular SODs are only 45% and 55%, respectively. Inhibitors of *Taenia solium* SOD, which potentially target a widely non­conserved cleft close to the dimerization interface, were docked to *Ov*EC-SOD. The overall structure of *Ov*EC-SOD and its dimerization were verified in solution. Structural insights into *Ov*EC-SOD may shed light on the structural diversity of SODs and may potentially further be exploited in future drug-discovery approaches to treat onchocerciasis with improved specificity.

## 2. Materials and methods

### 2.1. Cloning, protein production and purification

The open reading frame encoding *Ov*EC-SOD starting from Ala44 was amplified from *O. volvulus* genomic DNA by PCR (Table 1 ▸). The N-terminus of the protein is supplemented with an OmpA signal peptide mediating secretion to the periplasmic space, a Strep-tag II and a TEV protease cleavage site (ENLYFQ↓G). *Escherichia coli* BL21 (DE3) cells were transformed and grown in Luria–Bertani medium containing 50 µg ml^−1^ ampicillin at 310 K. When the optical density at 600 nm reached approximately 0.6, overexpression was induced by adding 200 µg anhydrotetracycline per litre of shaking culture. After 4 h, the cells were harvested by centrifugation and resuspended in lysis buffer (50 m*M* Tris–HCl, 500 m*M* NaCl, 5% glycerol pH 8.0). The cells were disrupted by sonication and the supernatant was loaded onto a StrepTactin Sepharose column (IBA, Germany) pre-equilibrated with lysis buffer. Protein was eluted using 2.5 m*M* desthiobiotin in the same buffer. The Strep-tag II was cleaved off by TEV protease at a molar ratio of 1:50 using the combined fractions containing *Ov*EC-SOD. Subsequent size-exclusion chromatography allowed estimation of the oligomeric state of *Ov*EC-SOD using a calibrated HiLoad 16/600 Superdex 200 column equilibrated with 50 m*M* Tris–HCl, 200 m*M* NaCl pH 8.0. The homogeneity and the optimal solution composition were verified by dynamic light scattering using a Spectrolight 300 instrument (XtalConcepts, Germany) in preparation for crystallization experiments. The identity and integrity of the purified protein were confirmed by SDS–PAGE (Laemmli, 1970 ▸) as shown in Supplementary Fig. S1(*a*).

### 2.2. Crystallization and crystal handling

*Ov*EC-SOD was initially screened against 400 distinct crystallization conditions (Qiagen, Germany) applying the sitting-drop vapor-diffusion method using a Honeybee 961 dispensing robot (Genomic Solutions, UK) at 293 K combined with a 2-well MRC plate. A 500 nl droplet of protein solution was mixed with the same volume of reservoir solution and equilibrated against 50 µl reservoir solution. Brick-shaped crystals appeared after one week using a reservoir solution consisting of 0.01 m*M* calcium chloride, 0.1 m*M* sodium acetate pH 4.0, 60%(*w*/*v*) MPD. Unfortunately, these crystals only diffracted to approximately 6 Å resolution. To optimize the crystal quality, the N-terminal tag was cleaved off and otherwise identical crystallization experiments were performed. Crystals appeared using the conditions specified in Table 2 ▸ and grew to full size after five months. The crystal morphology is shown in Supplementary Fig. S1(*b*).

Crystals were prepared for data collection by manual harvesting using nylon loops. They were briefly immersed in mother liquor supplemented with 10% glycerol as a cryoprotectant and were flash-cooled in liquid N_2_ for subsequent data collection.

### 2.3. Data collection, processing and refinement

Diffraction data were collected on EMBL beamline P13 at DESY, Hamburg, Germany. Further details are specified in Table 3 ▸. Indexing of the data was carried out with *XDS* (Kabsch, 2010 ▸). The crystal structure was solved by molecular replacement with *MOLREP* (Vagin & Teplyakov, 2010 ▸) using the crystal structure of a *C. elegans* SOD (PDB entry 3kbe; O. N. Pakhomova, A. B. Taylor, J. P. Schuermann, V. L. Culotta & P. J. Hart, unpublished work) as a search model. *REFMAC*5 (Kovalevskiy *et al.*, 2018 ▸) from the *CCP*4 suite version 4.2 (Winn *et al.*, 2011 ▸) was used for iterative refinement in combination with *Coot* (Emsley *et al.*, 2010 ▸) for manual model building. Model building resulted in an overall *R* of 15.9% and *R*_free_ of 18.1% using all data in the resolution range 25.80–1.55 Å. Data-collection, indexing and refinement statistics are shown in Tables 3 ▸ and 4 ▸, respectively. The structure was deposited in the Protein Data Bank with PDB code 5in2.

### 2.4. Structural investigation of *Ov*EC-SOD in solution

Monodisperse solutions containing pure *Ov*EC-SOD were applied to small-angle X-ray scattering (SAXS) to verify the dimerization of *Ov*EC-SOD and to analyze its shape. Data were collected on EMBL beamline P12 at the PETRA III storage ring, DESY, Hamburg, Germany as further specified in Supplementary Table S1. Four solute concentrations in the range 0.5–7.5 mg ml^−1^ were exposed to the beam. The obtained scattering amplitudes were averaged over all 40 exposures per sample for 45 ms each and the averaged buffer scattering of 40 buffer exposures was subtracted. Data were normalized to the transmitted beam intensity. The scattering profiles were plotted and evaluated using *PRIMUS* as part of the *ATSAS* suite (Manalastas-Cantos *et al.*, 2021 ▸). The Guinier approximation (Guinier, 1939 ▸) was utilized to determine the radius of gyration (*R*_g_). The pair distance-distribution function was calculated using *GNOM*. *Ab initio* models were calculated using *GASBOR* (Svergun *et al.*, 2001 ▸). Furthermore, *CRYSOL* (Svergun *et al.*, 1995 ▸) and *SREFLEX* (Panjkovich & Svergun, 2016 ▸), which considers additional conformational flexibility of a given high-resolution structure in solution, allowed the comparison of the scattering data to known high-resolution crystal structure coordinates.

### 2.5. Docking

Using *FTmap* (Kozakov *et al.*, 2015 ▸), potential binding sites for the default docking library of small molecules were identified. These binding sites and the individual pattern of binding sites on the surface of related SODs are considered to be useful in preparation for fragment-based drug-development approaches and also for identifying potential binding sites of a given ligand. The underlying probe library consistes of 16 molecules, *i.e.* acetamide, acetonitrile, acetone, acetaldehyde, methylamine, benzaldehyde, benzene, isobutanol, cyclohexane, *N*,*N*-dimethylformamide, dimethyl ether, ethanol, ethane, phenol, 2-propanol and urea.

The compounds for the docking of putative inhibitors were selected based on a previous docking study as well as activity and specificity assays on *T. solium* Cu/Zn-SOD (García-Gutiérrez *et al.*, 2011 ▸). These authors utilized the LeadQuest library, which contains 51 068 drug-like compounds, reduced the size of the screen using Lipinski-like rules for docking and finally tested 50 candidate compounds *in vitro*. The molecular weights of the library compounds range from 200 to 700 Da. Based on this previous study, which indicated inhibitor binding outside the active site (García-Gutiérrez *et al.*, 2011 ▸), and agreement with *FTmap* indicating potentially druggable binding sites, the *in silico* docking analysis was prepared. *AutoDock* 4.2.3 (Morris *et al.*, 2009 ▸) was used for compound docking to the *Ov*EC-SOD dimer applying the Lamarckian genetic algorithm (LGA). For each docking, the grid size was set to 60 × 60 × 60 Å with a grid spacing of 0.675 Å centered at the putative hydrophobic compound binding cleft in proximity to the dimerization interface of the *Ov*EC-SOD dimer [grid center coordinates (*x*, *y*, *z*): −9.3, −3.8, −13.5]. Step sizes of 1 Å for translation and 60° for rotation were chosen, the maximum number of energy evaluations was set to 150 000 and 150 runs were performed. Ligand-binding site plots were prepared using *LigPlot*+ version 2.2 (EMBL–EBI).

## 3. Results and discussion

### 3.1. Overall crystal structure

**Figure 1:**
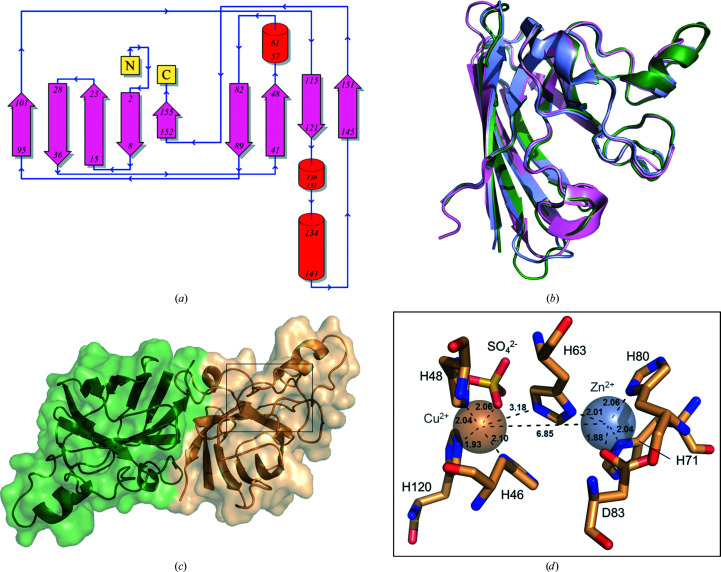
High-resolution crystal structure of *Ov*EC-SOD. (*a*) Topology diagram of the protein and its secondary structure. (*b*) Superposition of *O. volvulus* SOD (green) with *T. solium* SOD (pink; PDB entry 3mnd; C^α^ r.m.s.d. of 0.5 Å; Hernández-Santoyo *et al.*, 2011 ▸) and *H. sapiens* SOD (purple); PDB entry 1hl4; C^α^ r.m.s.d. of 0.5 Å; Strange *et al.*, 2003 ▸). (*c*) Cartoon and surface representation of the crystallographic *Ov*EC-SOD dimer. The approximate position of the active site as highlighted in (*d*) is framed. (*d*) Close-up of the active site and the two conserved metal ions. The coordination distances are shown in Å.

The major differences among Cu/Zn-type SODs at the sequence level are the length and composition of the large non­conserved turns and loops, as visualized as an alignment in Supplementary Fig. S2. The crystal structure of *Ov*EC-SOD contains one molecule per asymmetric unit; the respective data processing is summarized in Table 4 ▸. The biological assembly, however, is a dimer with two distinct independent active sites. Every monomer contains three α-helices, accounting for approximately 9% of the secondary structure, and nine β-sheets representing 37% of the secondary structure. The *Ov*EC-SOD structure shows typical features of SODs that are highly conserved throughout the different kingdoms of organisms, including the Greek-key β-barrel motif (Fig. 1 ▸ *a*). Closely related homologues from *Homo sapiens* share an overall r.m.s.d. of <1 Å for C^α^ positions with *Ov*EC-SOD (Fig. 1 ▸ *b*). The common feature is a large cylindrical barrel comprising nine extended sheets with an entirely antiparallel structure. The rest of the protomer structure contains two loops of a nonrepetitive type. The first loop, *i.e.* residues 47–84, has two distinct parts. The first part is a disulfide loop, which is connected to the β-barrel motif by a disulfide bond. The second part is predominantly hydrophilic and contributes to coordination of the Zn^2+^ ion. The second, and also the second largest, loop is hydrophilic and is referred to as the electrostatic loop. The dimerization interface mainly consists of hydrophobic interactions, involving only four hydrogen bonds. The dimer, as displayed in Fig. 1 ▸(*c*), has a dimerization interface area of approximately 730 Å^2^.

### 3.2. The active site of *Ov*EC-SOD and its geometry

The active site contains one Cu^2+^ ion relevant for catalysis and one structural Zn^2+^ ion. The identity of the metal ions is supported by the *CMM* server (Zheng *et al.*, 2014 ▸) based on their coordination geometries. Specifically, *B* factors of 21.4 and 18.3 Å^2^ were determined for the copper and zinc ions, respectively. Further, the gRMSD, *i.e.* the determined deviation from ideal coordination angles (ligand–metal–ligand) for the respective metal ion (Zheng *et al.*, 2014 ▸), is 9.4° for the copper ion and 7.9° for the zinc ion. The coordination spheres of the Cu^2+^ and Zn^2+^ ions are defined by the invariant residues His46, His48, His63 and His120 and His63, His71, His80 and Asp83, respectively (Fig. 1 ▸ *d*). The Zn^2+^ ion is coordinated by three histidine residues and one aspartate residue in a tetragonal geometry. The Cu^2+^ ion is coordinated by three histidine residues arranged in a distorted tetrahedral geometry. Additionally, many high-resolution SOD structures possess a water molecule that is involved in the coordination. To our knowledge, for the first time in a Cu/Zn-SOD structure a larger anion consisting of multiple atoms was identified in this position; although, it is generally not easy to crystallo­graphically distinguish sulfate from phosphate. The sulfate ion interacts via one of its O atoms, which is at a distance of 2.06 Å from the Cu^2+^ ion and appears to displace water (Fig. 1 ▸ *d* and Supplementary Fig. S3). As MOPS and HEPES are sulfonic acids, and sulfate (and also phosphate) was not part of the original crystallization solution and buffer composition, one can consider that the sulfate electron density might be part of a disordered buffer molecule or could alternatively originate from the *E. coli* culture or the purification environment. The copper ion is not only involved in the redox cycle of the catalytic activity but along with the zinc ion also contributes to the stability of the typical SOD β-barrel motif (Assfalg *et al.*, 2003 ▸). Hence, it might be hypothesized that the observed ligand ion of the complex could stabilize the protein at an elevated stress level in *E. coli*, although the requirements for formation of the copper complex are not fully understood *in vivo*. In human SOD1 in *E. coli* cells copper was reported to be absent in the context of NMR experiments (Banci *et al.*, 2011 ▸).

The catalytic activity of *Ov*EC-SOD seems to be independent of the presence of a conserved water molecule (Banci *et al.*, 1989 ▸). In *Ov*EC-SOD, His63 forms a ‘bridge’ between the Cu^2+^ and Zn^2+^ ions, with distances of 3.2 and 2.0 Å, respectively. The Cu–His63–Zn imidazolate bridge is intact in the oxidized form of the enzyme. In the reduced form of the enzyme this bond is broken and the catalytic metal becomes three-coordinated (Ascone *et al.*, 1997 ▸). In the oxidized form, the typical distance between copper and zinc should be approximately 6.0 Å, while in the reduced form of the enzyme this distance should be around or greater than 6.5 Å. The crystal structure of *Ov*EC-SOD revealed that this bond was broken at a distance of 6.9 Å between the metal ions (Fig. 1 ▸ *d*).

The active-site channel is formed in part by the disulfide loop, *i.e.* residues 48–62, and in part by the electrostatic loop, *i.e.* residues 130–146. The latter provides the electrostatic potential to drive the substrate to the reaction site with a major regulatory potential depending on charge and conformation (García-Gutiérrez *et al.*, 2011 ▸). However, the entrance of the channel to the active site varies in sequence and also in structure when comparing *Ov*EC-SOD with the homologous human enzyme. In human Cu/Zn-SOD this electrostatic loop is positively charged overall, while *Ov*EC-SOD contains more nonpolar and negatively charged residues. In *Ov*EC-SOD the loop is ordered, with well defined 2*F*_o_ − *F*_c_ electron density. The active-site channel is maintained by the conserved intramolecular disulfide bond. The side chain of Arg146, which is the residue responsible for the correct orientation of superoxide in the catalytic cavity and is highly conserved, is stabilized by hydrogen bonding to the carbonyl O atom of Cys57.

### 3.3. SAXS

**Figure 2:**
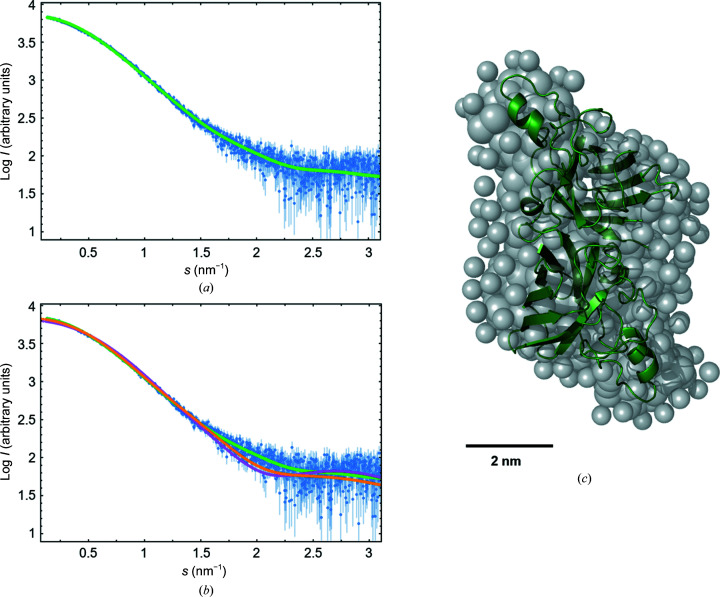
Solution structure of *Ov*EC-SOD. (*a*) Averaged normalized scattering intensities (blue) plotted against the momentum transfer *s* as obtained by SAXS. The calculated fit curve (green) is a comparison with the *ab initio* model shown as gray spheres in (*c*). (*b*) Scattering intensities and fit curve as shown in (*a*) superposed with additional fit curves of the *Ov*EC-SOD dimer crystal structure with the SAXS data using either *CRYSOL* (magenta) or *SREFLEX* (yellow) for structural comparison. (*c*) Superposition of the *Ov*EC-SOD dimer crystal structure in green and the *ab initio* model of *Ov*EC-SOD based on the SAXS data.

Monodisperse solutions containing purified *Ov*EC-SOD after affinity-tag cleavage were used in SAXS measurements to confirm the crystallographic dimer and analyze the solution structure (Fig. 2 ▸). There was no indication of concentration-dependent oligomerization. The scattering amplitudes recorded at different concentrations were averaged, resulting in the scattering pattern displayed in Fig. 2 ▸(*a*). SAXS data-collection parameters and characteristics of the protein are summarized in Supplementary Table S1.

The molecular mass of 38 ± 2 kDa was estimated from the forward scattering using bovine serum albumin as a reference protein. This approximation indicates that *Ov*EC-SOD is dimeric in solution. An experimental *R*_g_ of 2.55 ± 0.01 nm and a *D*_max_ of 9 ± 1 nm were determined. The *ab initio* structure was calculated using scattering vectors up to *s* = 0.4 Å^−1^ and indicated a rhomboidal shape that was superposed with the crystal structure. The fit curve of this *ab initio* model is shown in Fig. 2 ▸(*a*). Comparison of the experimental SAXS scattering data with theoretical scattering curves calculated using the crystal structure of the *Ov*EC-SOD dimer resulted in the optimized fit curves shown in Fig. 2 ▸(*b*). Considering the additional conformational flexibility of *Ov*EC-SOD in solution, the experiment confirms that the *Ov*EC-SOD dimer seen in the crystal resembles the overall structure of the protein in solution well. The core of the solution structure is formed by the presumably more rigid β-sheets surrounded by loop regions and helices at both ends of the elongated dimer, rendering the *ab initio* structure shown in Fig. 2 ▸(*c*). The scattering data including the described fitting is available in the Small Angle Scattering Biological Data Bank (SASBDB) as entry SASDPF2.

According to Muñoz *et al.* (2005 ▸), the dimerization of SODs in solution is typical and vital for their catalytic function. Dimer-destabilizing mutations of Cu/Zn-SODs have been related to a number of degenerative motor neurone diseases (Téllez-Valencia *et al.*, 2004 ▸; Hough *et al.*, 2004 ▸), with a very complex maturation pathway of the dimeric functional holo-enzyme, as recently summarized for SOD1 (Trist *et al.*, 2021 ▸).

### 3.4. Previously identified inhibitors and their potential binding sites

50 compounds from the LeadQuest library were selected *in silico* and screened *in vitro* for inhibition of *T. solium* SOD in a previous approach (García-Gutiérrez *et al.*, 2011 ▸). A set of polyaromatic compounds within this library showed inhibition of *T. solium* SOD *in vitro*, with IC_50_ values in the micromolar range. They were predicted to bind outside the highly conserved active site with specificity for *T. solium* SOD over the human homologue by *in silico* docking (García-Gutiérrez *et al.*, 2011 ▸). Docking scores are summarized in Supplementary Table S2. Since the residues directly or indirectly involved in metal binding are conserved among Cu/Zn-SODs, binding outside the narrow core active site in regions with lower sequence conservation is of particular interest for the development of highly species-specific SOD inhibitors. García-Gutiérrez and coworkers searched for potentially druggable binding cavities *in silico*, which may indicate an option to interfere with the catalytic activity of *T. solium* SOD. Similarly, we considered *in silico* predictions provided by the *FTmap* server utilizing small-molecule docking across the entire protein surface. *FTmap* predicted a wide central cleft, which is formed by both protomers of the *Ov*EC-SOD dimer, as a ‘hotspot’ for interaction with small molecules from the default *FTmap* library. The patterns of small-molecule interaction are notably different for the three homologue proteins that were analyzed despite the similarity of the proteins (Supplementary Fig. S4). This wide cleft spanning halfway around the narrow side of the dimer close to the dimerization site overlaps with a binding site considered and previously used in docking studies by García-Gutiérrez and coworkers. Utilizing this cleft as the definition of the region of interest, three of the previously analyzed *T. solium* Cu/Zn-SOD inhibitors were selected for a similar docking approach with *Ov*EC-SOD. Specifically, the targeted core of this cleft comprises amino acids 62–64, 104–113 and 152 of the *Ov*EC-SOD structure. This location is separated from the active site and hence the docking does not interfere with the metal-coordination sites. García-Gutiérrez and coworkers hypothesize that one explanation for the observed inhibition could be local restrictions of loop movement in close proximity to the abovedescribed cleft, resulting in reduced substrate delivery to the active site upon compound binding.

**Figure 3:**
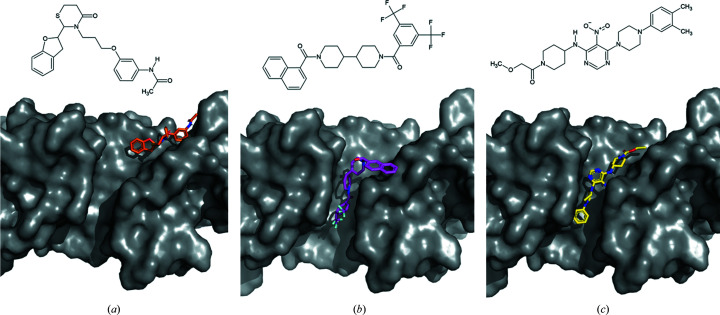
Docking of LeadQuest compounds to *Ov*EC-SOD (gray): (*a*) ID 1545-7806 (molecular mass 426.5 g mol^−1^; C_23_H_26_N_2_O_4_S), (*b*) ID 1460-00055 (molecular mass 562.6 g mol^−1^; C_30_H_28_F_6_N_2_O_2_), (*c*) ID 1502-3317 (molecular mass 438.6 g mol^−1^; C_77_H_87_F_6_N_11_O_10_S).

The three docked compounds are indicated to specifically interact with partly differing sections of the targeted cleft, as shown in Figs. 3 ▸(*a*)–3 ▸(*c*). The putative mechanism of binding is mainly assured through hydrophobic interactions and involves both protomers of the dimer, both of which are in agreement with the binding sites reported for *T. solium* SOD. A hydrogen bond to compounds 1545-7806 and 1502-3317 is formed via the backbone of the conserved Leu106 of *Ov*EC-SOD. Favoring species specificity, Arg3 and Arg107 of *Ov*EC-SOD, which contribute hydrophobic interactions with all three described ligands, as well as several amino-acid side chains between positions 107 and 115, which hydrophobically interact with at least one of the compounds, are not conserved. Previously, in the comparison of *T. solium* SOD and the homologue from *H. sapiens*, the partly nonconserved residues Thr107 and Ser111 were highlighted by García-Gutiérrez and coworkers; however, *T. solium* and *O. volvulus* share a serine at position 111. Detailed two-dimensional ligand-binding site plots for the docked compounds are displayed in Supplementary Fig. S5.

The docking scores obtained for *Ov*EC-SOD are slightly different from the values obtained for *T. solium* Cu/Zn-SOD, as summarized in Supplementary Table S2. However, for both homologues a higher affinity compared with the human SOD homologue is indicated *in silico*. A careful verification of these predictions by further *in vitro* experiments and by means of structural biology is nonetheless desirable, also considering the challenges in state-of-the-art docking approaches as summarized by Zev and coworkers in the context of a protease (Zev *et al.*, 2021 ▸). Further understanding of the mode of action of the compounds, potential off-target effects and options for allosteric specific inhibition of SODs would provide a solid perspective for structure-based optimization of the compounds based on the presented crystallographic data.

In summary, the widely conserved structure of *Ov*EC-SOD was solved and compared with its solution structure, which comprises an elongated dimer. Differences in metal-ion coordination are discussed. Based on the individual surface areas of *Ov*EC-SOD and related enzymes, initial suggestions for achieving species specificity in inhibitor development remain to be expanded.

## Supplementary Material

PDB reference: extracellular Cu/Zn superoxide dismutase, 5in2

SASBDB reference: extracellular Cu/Zn superoxide dismutase, SASDPF2

Supplementary Figures and Tables. DOI: 10.1107/S2053230X22005350/no5193sup1.pdf

## Figures and Tables

**Table 1:** Macromolecule-production information

Source organism	*Onchocerca volvulus* Leuckart, 1894
DNA source	Genomic DNA
Forward primer	5′-*GGGCAA* GAATTCCATGGCTAGAAGAGCAGTAGCAGT-3′
Reverse primer	5′-*GGGCAA* GGATCCTCAAGCAGCAATGCCAATAACACC-3′
Expression vector	pASK-IBA16 (IBA, Germany)
Expression host	*Escherichia coli* BL21 (DE3)
Complete amino-acid sequence	MKKTAIAIAVALAGFATVAQAASWSHPQFEKSGGGGGENLYFQGAETAVPNSMARRAVAVLRGDAGVSGIIYFQQGSGGSITTISGSVSGLTPGLHGFHVHQYGDQTNGCTSAGDHYNPFGKTHGGPNDRIKHIGDLGNIVAGANGVAEVYINSYDIKLRGPLSVIGHSLVVHANTDDLGQGTGNMREESLKTGNAGSRLACGVIGIAAVS

**Table 2:** Crystallization conditions for *Ov*EC-SOD

Method	Vapor diffusion, sitting drop
Plate type	96-well MRC2 plate
Temperature (K)	293
Protein concentration (mg ml^−1^)	18
Buffer composition of protein solution	50 m*M* Tris–HCl, 200 m*M* NaCl pH 8.0
Composition of reservoir solution	10%(*w*/*v*) PEG 20 000, 20%(*v*/*v*) PEG MME 550, 30 m*M* each of di-, tri-, tetra- and pentaethylene glycol, 0.1 *M* MOPS/HEPES–Na (premixed in an equimolar ratio) pH 7.5
Drop volume and mixing ratio	500 nl protein, 500 nl reservoir
Volume of reservoir (µl)	50

**Table 3:** Data collection and processing for *Ov*EC-SOD Values in parentheses are for the outer shell.

Diffraction source	Beamline P13, PETRA III
Wavelength (Å)	1.033
Temperature (K)	100
Detector	PILATUS 6M
Crystal-to-detector distance (mm)	180
Rotation range per image (°)	0.1
Total rotation range (°)	360
Exposure time per image (ms)	20
Space group	*P*3_1_21
*a*, *b*, *c* (Å)	58.4, 58.4, 77.6
α, β, γ (°)	90, 90, 120
Mosaicity (°)	0.22
Resolution range (Å)	25.80–1.55 (1.60–1.55)
Total No. of reflections	198412
No. of unique reflections	22806
Completeness (%)	100 (100)
Multiplicity	8.7 (8.0)
〈*I*/σ(*I*)〉	14.1 (3.33)
CC_1/2_	99.9 (99.9)
*R*_merge_	0.075 (0.420)
*R*_r.i.m._ †	0.07 (0.45)
Overall *B* factor from Wilson plot (Å^2^)	21.2
Matthews coefficient (Å^3^ Da^−1^)	2.4
Solvent content (%)	48.8

† Estimated as *R*_r.i.m._ ≃ *R*_merge_(*N*/(*N* − 1))^1/2^, where *N* is the data multiplicity.

**Table 4:** Structure solution and refinement for *Ov*EC-SOD Values in parentheses are for the outer shell.

Resolution range (Å)	25.80–1.55 (1.60–1.55)
Completeness (%)	100 (100)
No. of reflections, working set	22776 (2109)
No. of reflections, test set	1185 (122)
Final *R*_cryst_ (%)	15.9 (20.2)
Final *R*_free_ (%)	18.1 (27.7)
Cruickshank DPI	0.065
No. of non-H atoms	
Protein	1119
Ligands (including all ions)	27
Water	92
Total	1238
R.m.s. deviations	
Bond lengths (Å)	0.009
Angles (°)	1.240
Average *B* factors (Å^2^)	
Protein	29.6
Ligands	35.3
Water	35.6
Ramachandran plot	
Most favored (%)	98.3
Allowed (%)	1.7
