# Supplementary material for: Crystal structure of an extracellular superoxide dismutase from Onchocerca volvulus and implications for parasite-specific drug development
Source: Acta Crystallogr F Struct Biol Commun. 2022 May 27;78(Pt 6):232–40. doi: 10.1107/S2053230X22005350 (PMC9158661; doi:10.1107/S2053230X22005350)
Supplement: Supplementary file 1 [file f-78-00232-sup1.pdf]

Acta Cryst F

STRUCTURAL BIOLOGY COMMUNICATIONS

Volume 78 (2022)

Supporting information for article:

Crystal structure of an extracellular superoxide dismutase from *Onchocerca volvulus* and implications for parasite-specific drug development

Amr Moustafa, Markus Perbandt, Eva Liebau, Christian Betzel and Sven Falke

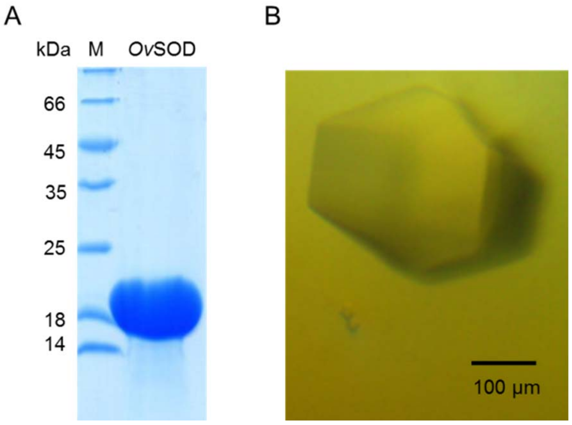

Figure S1 (**A**) Purified *Ov*EC-SOD analyzed by SDS-PAGE and used for robot-assisted crystallization trials. (**B**) Optimized polyhedral crystal of *Ov*EC-SOD.

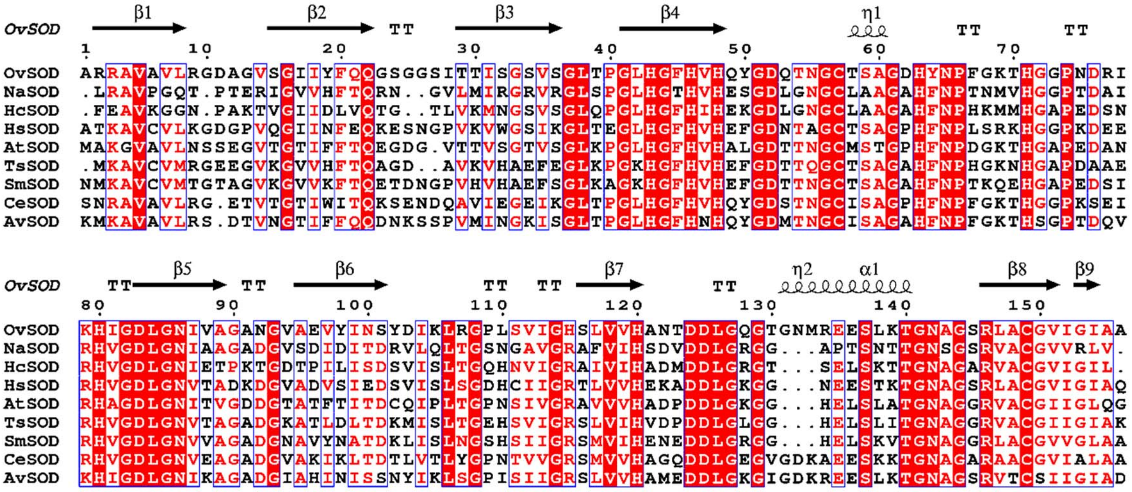

Figure S2 Sequence alignment of the *Ov*EC-SOD crystal structure (according to PDB: 5IN2) with distantly related Cu/Zn-SODs and the obtained secondary structure composition of *Ov*EC-SOD indicated on top. TT: β-turn, *Na*SOD: *Necator americanus* SOD (UniProt: W2TQI3), *Hc*SOD: *Haemonchus contortus* (UniProt: P51547), *Hs*SOD: *Homo sapiens* SOD1 (UniProt: P00441; PDB: 1HL4), *At*SOD: *Arabidopsis thaliana* SOD (UniProt: P24704), *Ts*SOD: *Taenia solium* SOD (UniProt: Q8WRF5; PDB: 3MND), *Sm*SOD: *Schistosoma mansoni* SOD (PDB: 1TO4_D), *Ce*SOD: *Caenorhabditis elegans* (UniProt: P34697 PDB: 3KBE), *Av*SOD: *Acanthocheilonema viteae* (UniProt: O77253).

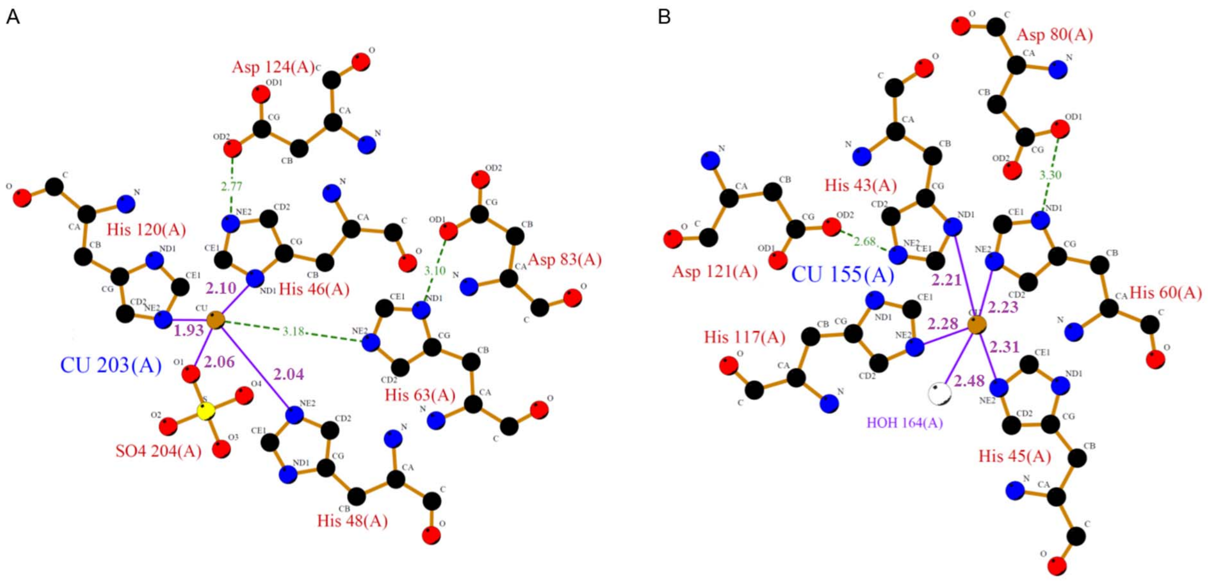

Figure S3 Comparison of the Cu ion coordination of (**A**) *Ov*EC-SOD and (**B**) *T. solium* SOD (PDB: 3MND, chain A) illustrated as two-dimensional plots according to PDBsum (https://www.ebi.ac.uk/thornton-srv/databases/cgi-bin/pdbsum/GetPage.pl?pdbcode=index.html; EMBL-EBI). Distances are provided in Å.

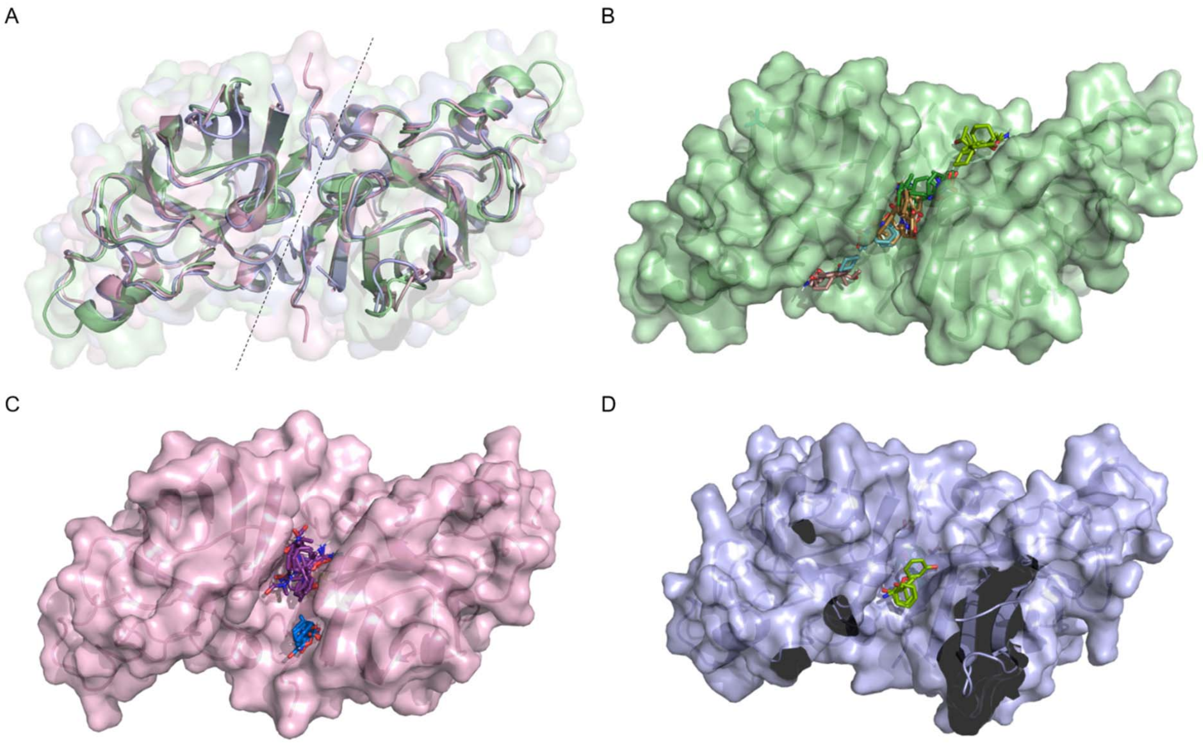

Figure S4 *In silico* identification of a potentially druggable surface cleft close to the dimer interface of *Ov*EC-SOD. (**A**) Superposition of dimers of *O. volvulus* (*green*) with *T. solium* (*pink*; PDB: 3MND; RMSD: 0.5 Å) and *H. sapiens* (*blue*; PDB: 1HL4; RMSD: 0.5 Å) SOD. The dimerization interface is schematically indicated by a dashed line. (**B**)-(**D**) FT map docking analysis across the surface of (**B**) *O. volvulus*, (**C**) *T. solium* and (**D**) *H. sapiens* SOD.

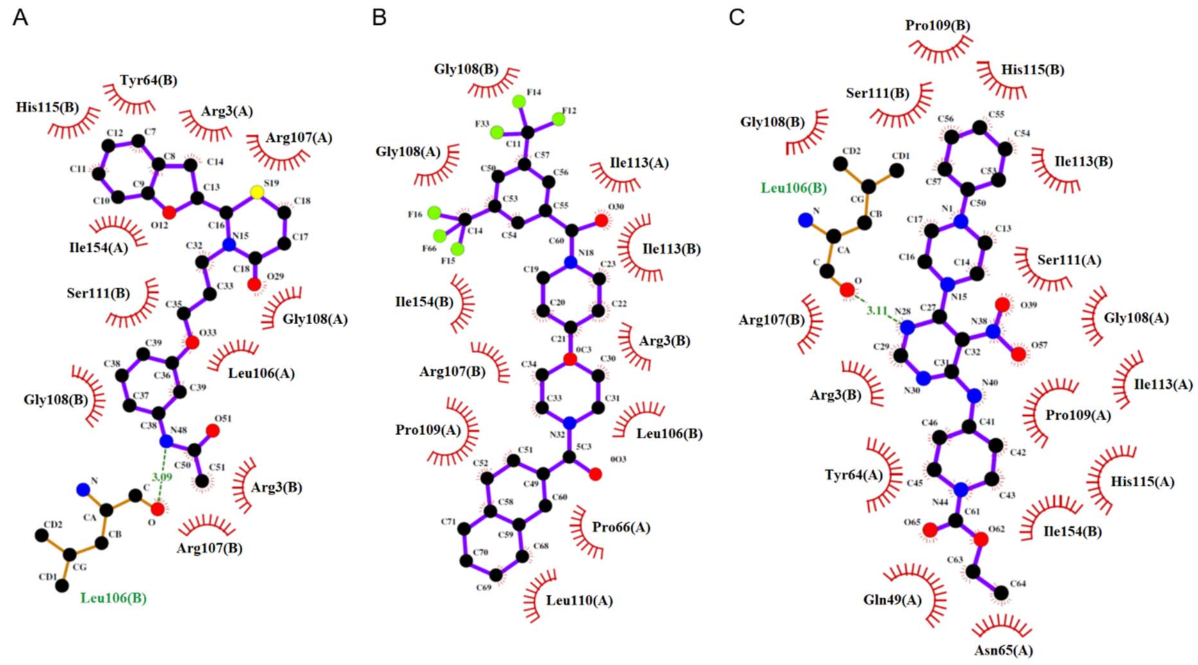

Figure S5 Two-dimensional plots of the compound interaction sites prepared using LigPlot+ and supplementing figure 3A-C: (**A**) compound ID *1545-7806*, (**B**) compound ID *1460-00055*, (**C**) compound ID *1502-3317*.

Table S1 SAXS data collection and dimensions of the *Ov*EC-SOD *ab initio* model (SASBDB accession code SASDPF2).

**Data collection**	
Synchrotron source	PETRA III
Beamline	EMBL beamline P12
Detector	2D photon counting Pilatus 2M pixel X-ray detector (Dectris, Switzerland)
Temperature (K)	293.15
Wavelength (nm)	0.124
Sample-detector distance (m)	3.0
Range of s (nm^{-1})	0.03 - 4.49
Exposure time	45 ms per exposure frame
Protein concentration (mg ml^{-1})	0.5 - 7.5
Buffer	50 mM Tris-HCl, 200 mM NaCl, 2 mM DTT, pH 8.0
**Dimensions of *Ov*EC-SOD**	
R_g (nm)	2.55 ± 0.01
D_{max} (nm)	9.0 ± 1.0
MW (based on volume of correlation V_c) (kDa)	32.2
MW (based on forward scattering I(0)) (kDa)	38 ±2
Volume [A^3]	37418

Table S2 Comparison of docking scores as obtained for selected compounds inhibiting *T. solium* Cu/Zn-SOD.

Compound	LeadQuestID	Best Score			*Ts*Cu/Zn-SOD IC_{50} (µM)[1]
		*Ov*SOD	*Ts*SOD[1]	*Hs*SOD[1]	
1	*1460-00055*	-8.1	-8.7	-6.0	23.9
2	*1502-3317*	-7.9	-9.0	-6.0	25.9
3	*1545-7806*	-7.6	-8.6	-5.5	- [2]

[1]García-Gutiérrez *et al*. (2011).

[2] Not specified exactly.
